# Supplementary material for: Phylogeography and Antioxidant Activity of Proso Millet (Panicum miliaceum L.)
Source: Plants (Basel). 2021 Oct 5;10(10):2112. doi: 10.3390/plants10102112 (PMC8537217; doi:10.3390/plants10102112)
Supplement: Supplementary file 1 [file plants-10-02112-s001.zip › Phylogeography and antioxidant activity of proso millet (Panicum miliaceum L.)-Supplementary Materials.pdf]

plants

MDPI

Supplementary Materials:

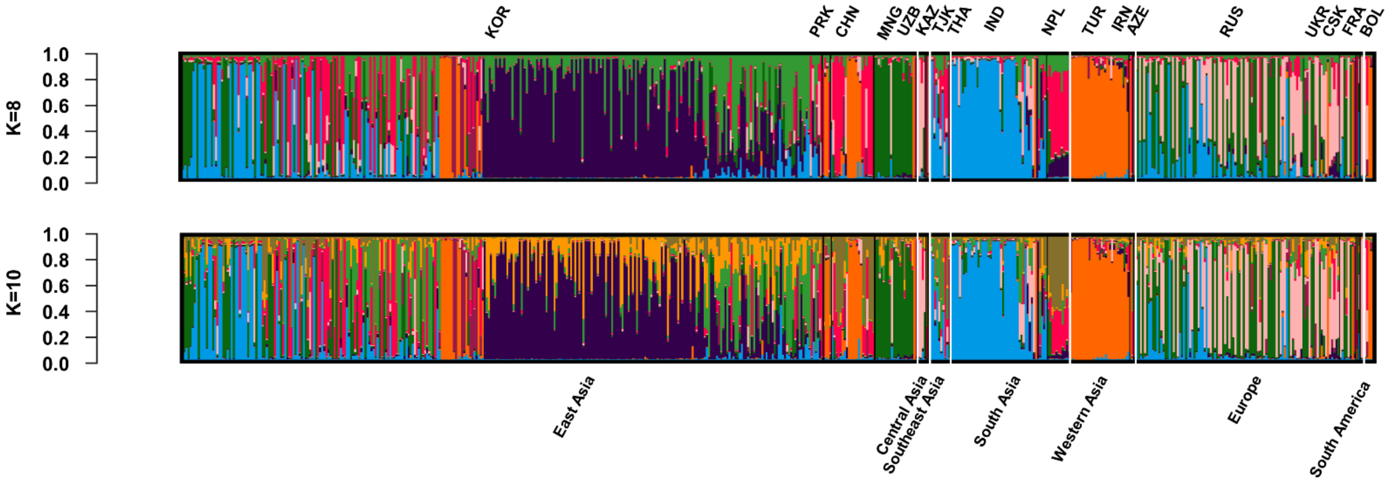

Figure S1. Population structure diagrams when the numbers of subpopulations (K) were 8 and 10.

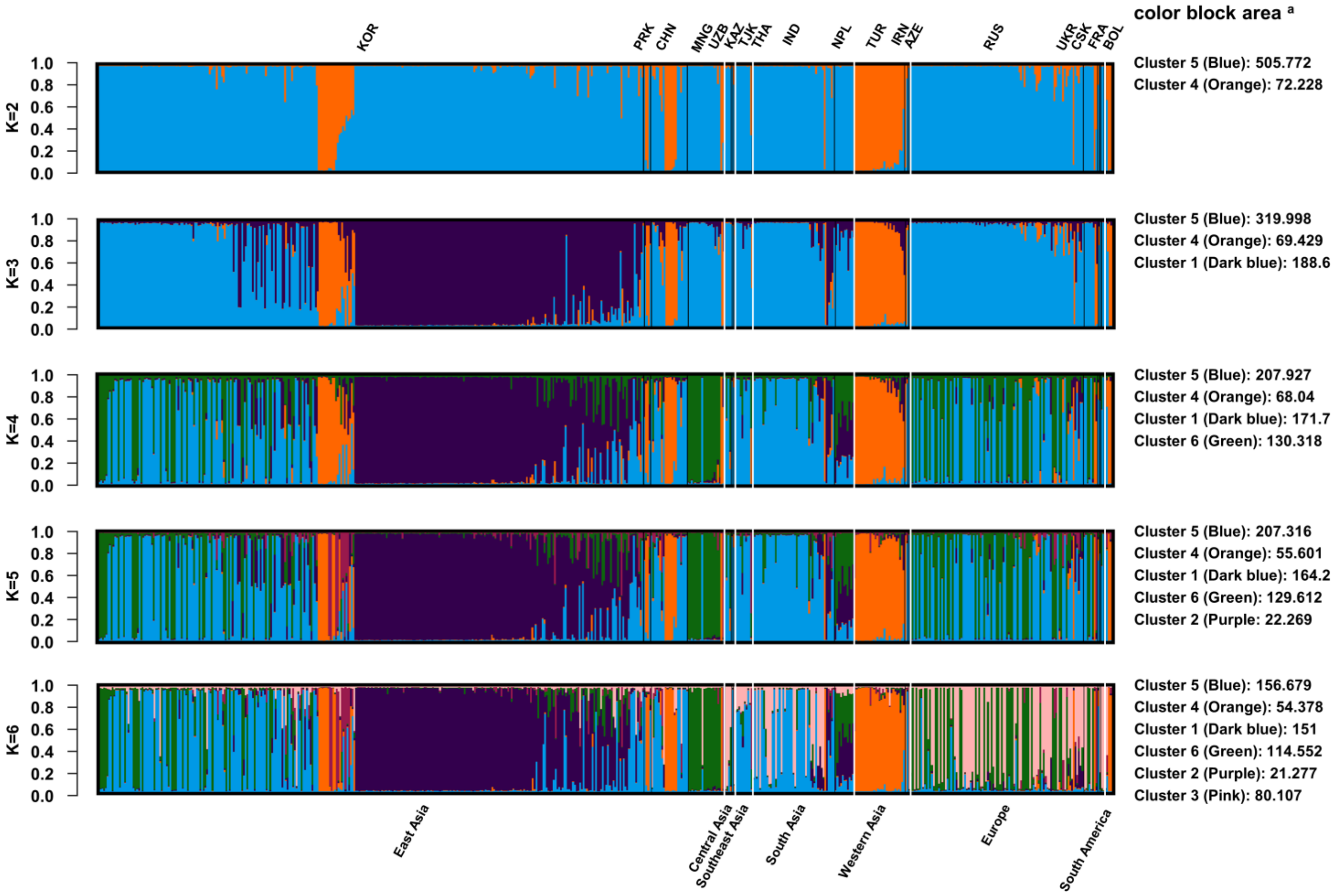

Figure S2. Trends in population structure as K increased from 2 to 6. [a] Colored area indicates proportions based on a total of 578 accessions.

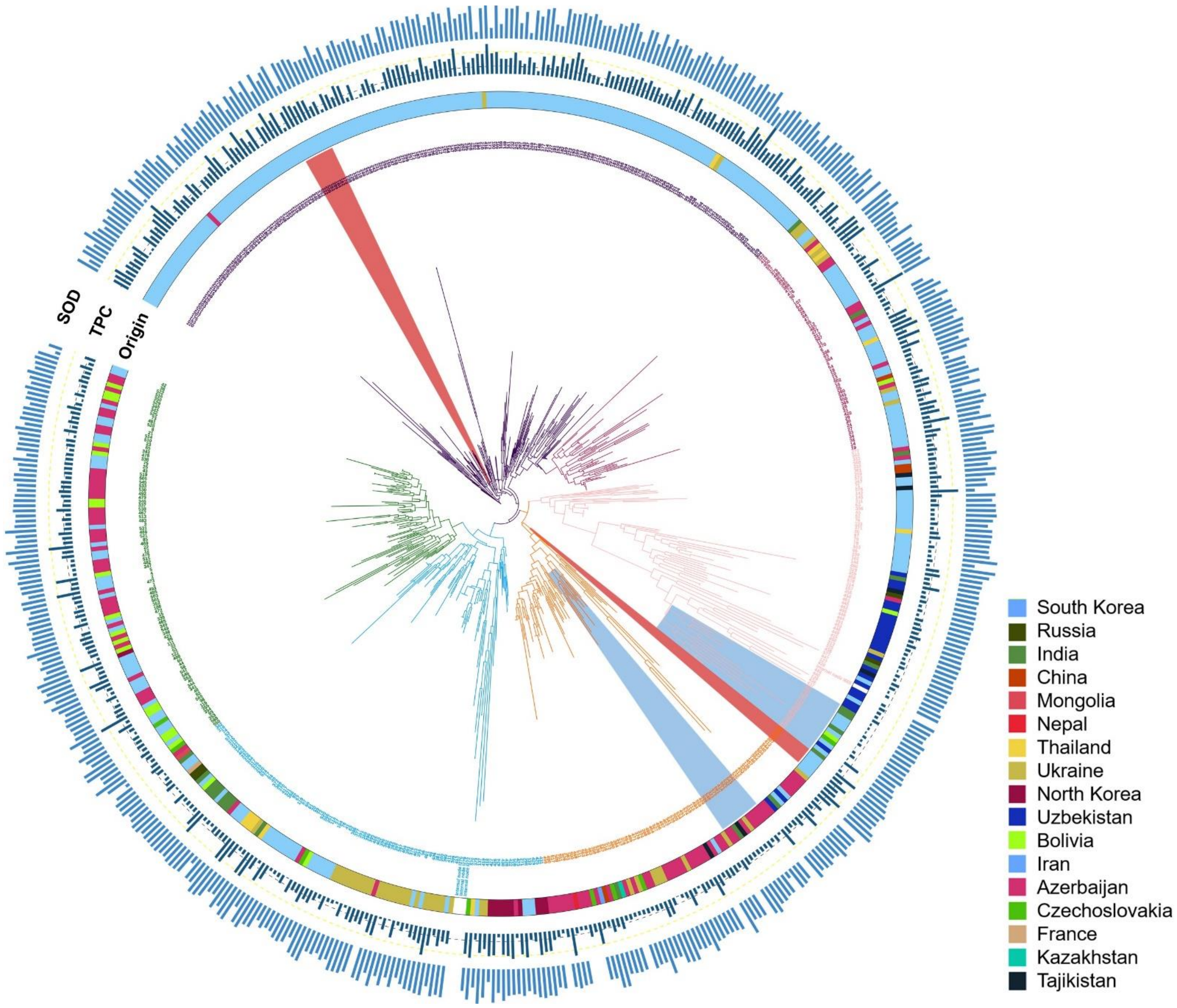

Figure S3. Phylogenetic analysis using 37 SSR markers and the neighbor-joining method. We compared the phenotypes of 578 individuals in six genotype clusters. Three groups of phenotypes were arranged in the outer circle of the phylogenetic tree in the form of a simple bar.

Table S1. Genetic diversity of each cluster and region.

**Cluster 1**		**Cluster 2**		**Cluster 3**		**Cluster 4**		**Cluster 5**		**Cluster 6**	
**Origin**	**Diversity**	**Origin**	**Diversity**	**Origin**	**Diversity**	**Origin**	**Diversity**	**Origin**	**Diversity**	**Origin**	**Diversity**
**KOR**	**0.138092**	KOR	0.086145	KOR	0.143174	KOR	0.154474	KOR	0.223160	**KOR**	**0.094595**
IND	0.103448	**CHN**	**0.162162**	CHN	0.143174	PRK	0.148649	PRK	0.013514	MNG	0.092444
		THA	0.024024	IND	0.143174	CHN	0.205185	**CHN**	**0.320863**	RUS	0.093454
		IND	0.037507	TUR	0.143174	UZB	0.040541	MNG	0.222973	UKR	0.040541
		RUS	0.118118	RUS	0.143174	**TUR**	**0.274163**	THA	0.131351		
				UKR	0.143174	RUS	0.156156	IND	0.079478		
								NPL	0.037162		

				RUS 0.092905	
				UKR 0.199324	

Table S2. Genetic diversity and combinations of six clusters.

Cluster	Gene diversity	Non polymorphic locus	Non polymorphic locus number
1	0.1396	SSR-67 (220/220), SSR-70 (132/132), SSR-82 (335/335), SSR-120 (224/224), SSR-121 (183/183), SSR-142 (124/124), SSR-143 (144/144), SSR-146 (177/177)	8
2	0.0992	SSR-71 (191/191), SSR-121 (183/183), SSR-131 (349/349), SSR-143 (144/144), SSR-420 (214/214), SSR-448 (259/259), SSR-458 (208/208), SSR-460 (270/270)	8
3	0.1432	SSR-100 (236/236), SSR-128 (263/263), SSR-131 (349/349), SSR-142 (124/124), SSR-146 (177/177), SSR-404 (302/302), SSR-420 (214/214), SSR-448 (259/259), SSR-460 (270/270)	9
4	0.2484	SSR-460(270/270)	1
5	0.1746	-	-
6	0.0992	SSR-31 (287/287), SSR-67 (220/220), SSR-70 (132/132), SSR-82 (335/335), SSR-85 (363/363), SSR-109 (222/222), SSR-120 (224/224), SSR-128 (263/263), SSR-129 (239/239), SSR-131 (349/349), SSR-142 (124/124), SSR-143 (144/144), SSR-404 (302/302), SSR-420 (214/214), SSR-430 (194/194), SSR-448 (259/259), SSR-458 (208/208), SSR-460 (270/270)	18
